# Impact of a Model Used to Simulate Chronic Socio-Environmental Stressors Encountered during Spaceflight on Murine Intestinal Microbiota

**DOI:** 10.3390/ijms21217863

**Published:** 2020-10-23

**Authors:** Corentine Alauzet, Lisiane Cunat, Maxime Wack, Laurence Lanfumey, Christine Legrand-Frossi, Alain Lozniewski, Nelly Agrinier, Catherine Cailliez-Grimal, Jean-Pol Frippiat

**Affiliations:** 1 Stress Immunity Pathogens unit (SIMPA), EA 7300, Université de Lorraine, F-54000 Nancy, France; lisiane.cunat@univ-lorraine.fr (L.C.); christine.frossi@univ-lorraine.fr (C.L.-F.); alain.lozniewski@univ-lorraine.fr (A.L.); catherine.cailliez@univ-lorraine.fr (C.C.-G.); jean-pol.frippiat@univ-lorraine.fr (J.-P.F.); 2 CHRU-Nancy, Service de Microbiologie, F-54000 Nancy, France; 3 Département d’Informatique Médicale, Biostatistiques et Santé Publique, Hôpital Européen Georges Pompidou, Assistance Publique-Hôpitaux de Paris, 75015 Paris, France; maximewack@free.fr; 4 Centre de Recherche des Cordeliers, INSERM, UMRS 1138, Université de Paris, 75006 Paris, France; 5 Institute of Psychiatry and Neuroscience of Paris (IPNP), INSERM U1266, Université de Paris, F-75014 Paris, France; laurence.lanfumey@inserm.fr; 6 CHRU-Nancy, INSERM, Université de Lorraine, CIC, Epidémiologie Clinique, F-54000 Nancy, France; nelly.agrinier@univ-lorraine.fr

**Keywords:** gut microbiota, chronic unpredictable mild stress, spaceflight, *Barnesiella*

## Abstract

During deep-space travels, crewmembers face various physical and psychosocial stressors that could alter gut microbiota composition. Since it is well known that intestinal dysbiosis is involved in the onset or exacerbation of several disorders, the aim of this study was to evaluate changes in intestinal microbiota in a murine model used to mimic chronic psychosocial stressors encountered during a long-term space mission. We demonstrate that 3 weeks of exposure to this model (called CUMS for Chronic Unpredictable Mild Stress) induce significant change in intracaecal β-diversity characterized by an important increase of the *Firmicutes*/*Bacteroidetes* ratio. These alterations are associated with a decrease of *Porphyromonadaceae*, particularly of the genus *Barnesiella*, a major member of gut microbiota in mice and humans where it is described as having protective properties. These results raise the question of the impact of stress-induced decrease of beneficial taxa, support recent data deduced from in-flight experimentations and other ground-based models, and emphasize the critical need for further studies exploring the impact of spaceflight on intestinal microbiota in order to propose strategies to countermeasure spaceflight-associated dysbiosis and its consequences on health.

## 1. Introduction

Gut microbiota (GM) form a complex microbial ecosystem whose balance and homeostasis are essential to the well-being of the host. Its composition is affected by numerous intrinsic and extrinsic factors such as antibiotics or diet [1,2]. Recent works have shown that host stress, particularly chronic stress, also has profound effects on the composition and organization of GM [3,4,5,6,7,8,9]. Chronic or excessive stress could be involved in the onset or exacerbation of chronic disorders such as anxiety and depression, or intestinal bowel diseases (IBD) [10]. More and more studies suggest a link between these pathologies and intestinal dysbiosis [2,10,11,12,13]. The sympathetic nervous system and the hypothalamic–pituitary–adrenal (HPA) axis represent the main biological stress axes and are strongly involved in the bidirectional communication between the gut and the central nervous system, also called brain gut axis [7,8,13,14,15]. This could explain how host stress impacts intestinal bacteria. Stress-induced modulation of GM could also be explained by the alteration of local immunity, intestinal motility, mucin secretion or visceral perception [5,16]. Furthermore, stress mediators released in the lumen, such as glucocorticoids and catecholamines, can directly modulate bacterial growth, virulence and gene expression [6,14,17,18,19].

During spaceflight, astronauts face chronic or intermittent stressors of psychosocial (confinement, isolation, sleep deprivation, persistent circadian misalignment) or physical (gravitational changes, radiations) origins [20]. These stressors, associated with dysregulation of the immune system [21,22], put astronauts at high risk of developing intestinal dysbiosis as illustrated by a recent study on International Space Station crew members reporting alteration of the composition of astronauts’ microbiome during space travel [23]. Such dysbiosis could have an impact not only on immune system efficiency, but also on energy intake, nutriments assimilation and intermediary metabolisms such as those of antibiotics [24]. As imbalance in GM could be correlated with a shift from a healthy state to a diseased state, it is important to evaluate the status of GM in response to chronic stressors encountered during long-duration space missions [25].

**Figure 1:**
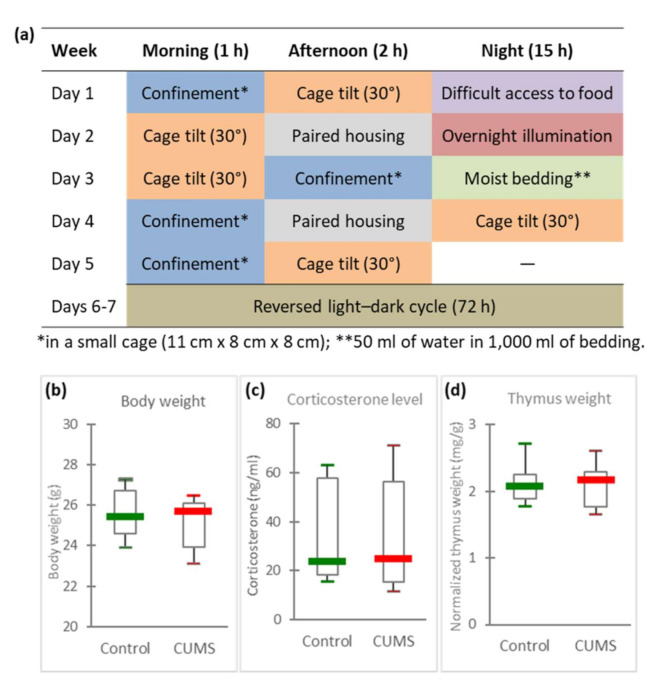
Stress status of mice. (**a**) Chronic Unpredictable Mild Stress (CUMS) protocol, (**b**) body weights, (**c**) serum corticosterone concentrations, (**d**) thymus weights normalized to body weight in control and CUMS mice. No statistically significant differences were found using the Mann–Whitney U test.

Given constraints imposed by in-flight experimentation and limited access to such an experimental platform, various ground-based models have been developed to reproduce the effects of spaceflight conditions on an organism. Using a ground-based model, we recently showed that certain conditions disrupt murine intestinal microbiota [3]. However, weight changes are not the only stressors encountered during space missions. Chronic socio-environmental factors such as confinement, circadian rhythm misalignment and psychosocial stressors have to be considered. Consequently, in this study, we used an easy-to-implement model (CUMS model, Table 1, Figure 1a), involving the chronic exposure of mice to multiple unpredictable mild environmental and psychosocial stressors, to simulate chronic socioenvironmental stresses encountered during a spaceflight and explore their effects on GM composition. We believe that this model comes reasonably close to the diversity and intensity of socio-environmental stressors encountered by astronauts while they are aboard the ISS, as shown in Table 1. Moreover, we previously showed that this model replicates some spaceflight-induced immunological changes observed in astronauts [26].

## 2. Results

### 2.1. Days of CUMS Exposure Do Not Induce a Major Stress Response

Male mice were divided in two groups: ten mice submitted to 21 days of CUMS and ten controls placed in another room of the animal facility. Animals presenting injuries, such as bites that could induce inflammation, were discarded resulting in ten CUMS mice and seven controls at the end of the experiment. To evaluate stress, mice were weighted at the end of the experimental procedure and the amount of corticosterone in peripheral blood was quantified by ELISA. Figure 1b,c show that these two parameters were similar in both groups of mice. We also determined thymus weight since it is well known that stress induces its involution. This organ weight was normalized to body weight (Figure 1d). Again, no statistically significant difference could be noted between the two groups of mice.

### 2.2. Intestinal Microbiome β-Diversity Is Significantly Modified by CUMS

**Figure 2:**
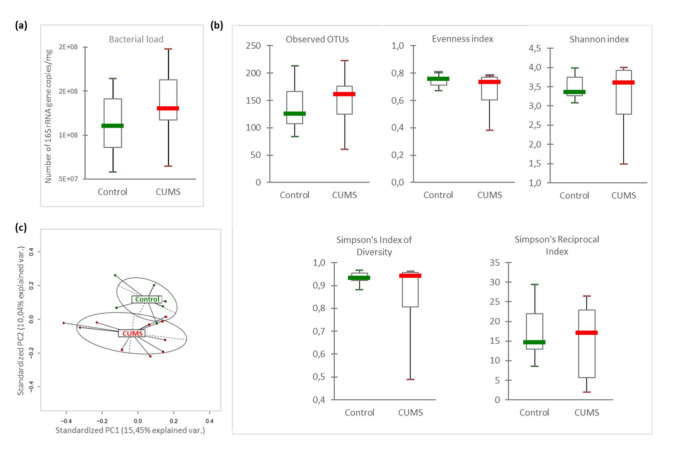
Comparison of microbiota diversity between CUMS and control mice. (**a**) Total bacterial load quantification by qPCR corresponding to the total number of 16S rRNA gene copies per mg of intracaecal content in mice subjected to CUMS (*n* = 10) and in control mice (*n* = 7) (*p* = 0.19). (**b**) α-diversity indexes: Observed OTUs (richness, *p* = 0.73), Evenness (*p* = 0.52), Shannon index (*p* = 0.84), Simpson index of diversity (*p* = 0.69), and Simpson’s reciprocal index (*p* = 0.81). Statistical analyses were done using the Mann-Whitney U test. The upper and lower ranges of the box represent the 75% and 25% quartiles, respectively. Error bars reflect standard error of the mean. (**c**) PCA of microbiomes from CUMS vs. control mice (*Pr(>F)* = 0.029). The variance explained by each of the main two dimensions of the PCA is indicated in parentheses on the axes.

To evaluate the effects of CUMS exposure on GM, we quantified by qPCR the number of 16S rRNA encoding gene copies per mg of intracaecal content. Figure 2a reveals that bacterial load was not significantly affected by CUMS exposure (CUMS: 1.12 × 10^8^ ± 1.34 × 10^7^ vs. controls: 1.39 × 10^8^ ± 1.67 × 10^7^, *p* = 0.19) (Figure 2a). We also preformed pyrosequencing experiments. They generated an average of 9024 reads per sample (ranging from 4276 to 24,502) with a mean length of 527 bp (ranging from 517 to 533 bp). Individual rarefaction curves (Supplementary Materials Figure S1) showed that the mean numbers of observed operational taxonomic units (OTUs), 140 taxa (ranging from 61 to 210 OTUs), reached in all samples a plateau of approximately 5000 sequence reads. The read coverage was therefore sufficient to capture most of the bacterial diversity of each intracaecal microbiome.

The within-sample diversity (α-diversity) indicated no significant difference between CUMS and control mice (Figure 2b). This suggests that CUMS mice had no change in microbial richness and evenness. However, in terms of β-diversity, Principal Component Analysis (PCA) showed distinct clustering between samples from control and CUMS mice indicating a significant change in microbiome composition (Figure 2c, PERMANOVA *p* = 0.029).

### 2.3. Impact on Caecal Microbiome Composition

A more in-depth taxonomic analysis of bacterial types revealed several changes in microbiome composition, and variations appeared at different phylogenetic levels. Nine divisions were identified by pyrosequencing. In all samples, the majority of caecal bacteria (ranging from 92 to 98% of total 16S) belonged either to the *Firmicutes* (ranging from 49.3 to 94.4%) or to the *Bacteroidetes* phylum (ranging from 2.5 to 46.8%), with a small proportion (2–8% of the identified sequences) of bacteria from seven others phyla: *Actinobacteria*, Candidatus *Melainobacteria*, Candidatus *Saccharibacteria* (TM7), *Cyanobacteria*, *Proteobacteria*, *Tenericutes* and *Verrucomicrobia* (Supplementary Materials Database S1). Moreover, 16 classes, 26 orders, 53 families, and 123 genera were identified.

**Figure 3:**
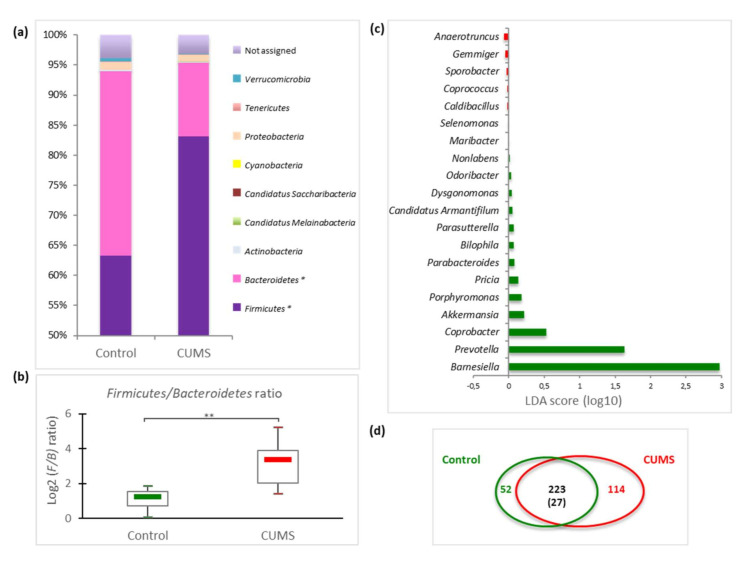
Differential abundance of bacterial taxa. (**a**) Mean relative abundance (%) of bacterial phyla and (**b**) *Firmicutes*/*Bacteroidetes* ratio in the caecal content of mice subjected to 21 days of CUMS compared to controls. Statistical analyses were done using the Mann-Whitney U test. * *p* < 0.01; ** *p* < 0.001. (**c**) Differentially abundant main genera in control mice (green) and CUMS mice (red) identified using Linear Discriminant Analysis (LDA) Effect Size (LEfSe) analysis. (**d**) Schematic representation of *core microbiome* at the species level. Green circle: number of species shared in all control mice. Red circle: number of species shared in all CUMS mice. Intersection and numbers inscribed within refer to shared species and in parentheses shared with all mice.

CUMS led to an increase of the *Firmicutes* phyla (*p* = 0.0041) and a decrease of the *Bacteroidetes* taxa (*p* = 0.0062) compared to control mice (Figure 3a).

These alterations induced a significant rise of the *Firmicutes*/*Bacteroidetes* ratio from 2.28 ± 0.38 in controls to 11.75 ± 3.43 in CUMS mice (Figure 3b, *p* = 0.00072). The gain of *Firmicutes* in CUMS mice was not clearly associated to the expansion of distinct genera, except for the *Clostridiales* members *Anaerotruncus*, *Coprococcus* and *Sporobacter* (Figure 3c), but seemed rather to be due to a general moderate rise of several taxa within the phylum. Concerning the diminution of *Bacteroidetes*, it is clearly linked to a significant decrease of *Porphyromonadaceae* (*p* = 0.022) and *Flavobacteriaceae* (*p* = 0.073) with the corresponding impacted genera being *Barnesiella*, *Prevotella*, *Coprobacter*, *Porphyromonas*, *Pricia*, *Parabacteroides*, *Dysgonomonas* and the vanishing of *Nonlabens* and *Maribacter* (Figure 3c, Supplementary Materials Database S1). We also noticed the lowering of another *Bacteroidetes* (Candidatus *Armantifilum* and *Odoribacter*) and of members of the genus *Akkermansia*.

At the species level, of the 389 taxa assigned, 275 species were found in control mice and 337 species in CUMS mice, corresponding to 223 species recovered in both groups (Figure 3d). Among them, only 27 were shared by all animals (*core microbiome*).

## 3. Discussion

It is increasingly evident that chronic psychosocial stresses influence intestinal homeostasis. Such alterations in microbiome composition can lead to local or central dysregulations that could be involved in the onset or exacerbation of chronic disorders such as IBD or psychiatric disorders [2,13,27,28]. During spaceflight, astronauts are subjected to various chronic physical and psychosocial stressors which could lead to dysbiosis, in a context of limited medical procedures and facilities. It has already been shown, using ground-based murine models, that weight modulation induces disruption of intestinal microbiota [3,29]. In this study, we used the CUMS model to mimic chronic socioenvironmental stresses encountered during space travels and explore their impact on intestinal microbiota. Indeed, we previously showed that this model replicates some spaceflight-induced immunological changes observed in astronauts [26]. Furthermore, it is recognized as a reliable and effective rodent model of depression [9,13,15,28,30,31,32].

Our results revealed that after 3 weeks of CUMS exposure, a duration chosen to simulate a six-month flight at the human scale [33], there was no significant change in murine caecal bacterial load. Additionally, no statistically significant modification of the α-diversity was observed in CUMS mice by comparison to controls, indicating that the within-community diversity was not altered by this model of chronic stress. Although these results are in agreement with other studies using variants of the rodent CUMS model [9,28], they are discrepant when compared to other works describing a decrease of α-diversity [11,15,31,32]. Such differences could be explained by variation in the CUMS models (species, strains, age, gender, feeding conditions, type of stressors, duration of exposure to individual stress), the origin of the samples (fecal or intraluminal), or protocols (DNA extraction method, PCR parameters) [3].

However, significant change in intracaecal global β-diversity was observed after CUMS treatment. Indeed, an important increase of the *Firmicutes*/*Bacteroidetes* ratio was observed in CUMS mice, which is consistent with other reports using variants of the rodent CUMS model [9,15,28,31]. Within the *Bacteroidetes* phylum, we observed a decrease of *Porphyromonadaceae* that has already been noted with other chronic stress such as restraint stress [34] and multifactorial model of early-life adversity [35]. Within this family, the greatest impact of CUMS was observed on the relative abundance of *Barnesiella* sp., a genus composed of *Barnesiella intestinihominis* and *Barnesiella viscericola*, belonging to the *core microbiome* of the murine and human gut. These species are described as having beneficial effects, such as protecting against colitis [36], enhancing the efficacy of antitumor treatments [37] and conferring resistance to intestinal colonization by pathogenic microorganisms [38]. These data raise the question of the impact of the decrease of this major member of GM in CUMS mice.

On the other hand, the increase of *Firmicutes* in CUMS mice cannot be statistically correlated with the increase of specific OTUs. This lack of correlation could be due to high interindividual variability in GM illustrated by the small number of species shared by all animals, stressed or not, suggesting the existence of only a reduced *core microbiome*. Such variability could also explain the lack of statistical significance at low taxa level and the fact that the impact of CUMS was manifest only at the phylum level. It is noteworthy that CUMS is associated with the appearance of several new taxa (114, Figure 3d), mainly belonging to *Firmicutes*, among them various OTUs of *Lactobacillus* with a great interindividual variability. Some protective taxa appeared (*Lactobacillus johnsonii*) while other decreased (*Lactobacillus murinus*), potentially offsetting each other. Interestingly, we observed opposite results when using a 3G-hypergravity model with a lowering of *L. johnsonii* and a rise of *L. murinus* [3]. Moreover, 3G-hypergravity was associated with increased bacterial load and α-diversity, as well as with a significant impact on the relative abundance of 50 intestinal species, whereas 2G-hypergravity seemed to modulate only moderately the GM composition. As described for the 2G-hypergravity model, the moderate alteration of GM observed with the CUMS model could be due to a lower activation of the HPA axis as no elevation of corticosterone level was noted in mice sera. This hypothesis is supported by higher serum corticosterone concentrations noticed in mice exposed to 3G during 21 days [39], as well as during the first two weeks of exposition to the chronic mild stress model (CMS) which is more intense than CUMS because of water and food deprivation periods [40]. So, as previously reported for the TCRβ repertoire [41,42], chronic socio-environmental stressors seem to have less impact on intestinal microbiota than gravity changes.

The results of the present study demonstrate that 3-weeks of exposure to chronic unpredictable psychosocial and environmental stressors alter mice GM, although to a lower extent than gravity changes. One limitation of this study is the small sample size that could lead to miss some modifications of GM because of intraindividual variability precluding their statistical detection. However, alteration of GM must receive attention and should be monitored in crewmembers, especially since it has been recently shown that a fecal transfer of GM from CUMS to healthy mice induces despair-like behaviors associated with alterations in serotonin pathway [32]. Furthermore, these data provide additional arguments to the countermeasure protocol proposed by experts against spaceflight-associated perturbations to the immune system [22]. Their recommendations include physical and psychological exercises for stress management, pre- or probiotics supplementation and dietary approaches, that could also permit to limit dysbiosis and its consequences on health. Finally, note that the results of this study go beyond astronaut health protection because the CUMS model can also be used to study the impact of everyday life stresses and it is well established that stress can contribute to the development or aggravation of several pathologies [2,43].

## 4. Materials and Methods

### 4.1. Experimental Animals and Ethics Statement

C57BL/6j male mice (8-week-old, mean body mass of 20 g) were purchased from Charles River (Les Oncins, France). On arrival, animals were housed for 5 days in groups of five in standard cages in the animal facility of the INSERM UMR 894 laboratory (Paris). They were housed in a quiet room under constant conditions (22 °C, 50% relative humidity, 12-h light/dark cycles with dark periods from 8 pm to 8 am) with free access to standard food and water. Then, mice were randomly divided in two groups housed in two separate rooms: one control group and one group subjected to CUMS for 21 days. Experimental procedures were carried out in conformity with the National Legislation and the Council Directive of the European Communities on the Protection of Animals Used for Experimental and Other Scientific Purposes (2010/63/UE). The CUMS protocol was approved by the French Ministry of Research (authorization 00966.02, approval date 24 January 2014).

### 4.2. Exposure to Chronic Unpredictable Mild Psychosocial and Environmental Stressors (CUMS Model)

Isolated animals (one mouse per cage) were subjected during 21 days to different unpredictable mild psychosocial and environmental stressors, according to Pardon et al. (2000) [44]. The CUMS procedure presented in Figure 1a was scheduled over a 1-week period and repeated throughout the 3 weeks of experimentation. Stress periods were always separated by stress-free intervals of at least 2 h to avoid any habituation process. The control group was left undisturbed in another room of the animal facility, five mice per standard cage (37.5 cm × 21.5 cm × 18 cm). Animals presenting injuries (such as bites that could induce inflammation) were discarded resulting in 7 control mice and 10 CUMS mice.

### 4.3. Sample Collection

At the end of the experiment, CUMS and control mice were anesthetized using isoflurane, weighed and then put to death by cervical dislocation. All samples were immediately processed to avoid degradation and/or contamination. The intestine was dissected in by excising the entire caecum. Samples were opened longitudinally and their contents were removed by two successive washes in DEPC (1‰)-treated PBS. Intra-luminal contents were immediately frozen in liquid nitrogen and stored at −80 °C until DNA isolation.

### 4.4. Corticosterone Quantification

Corticosterone was quantified in serum samples without any extraction procedure using the Corticosterone Enzyme Immunoassay kit (ArborAssays, Ann Arbor, MI, USA). Samples were analyzed in duplicate. Absorbance at 405 nm was measured and concentrations, calculated from a standard curve established using calibrators, were expressed as ng/mL.

### 4.5. DNA Isolation

Whole genomic DNA was extracted from caecal samples (50 mg) using the Fast DNA SPIN kit for Soil (MP Biomedicals, Santa Ana, CA, USA) [45] after bead beating with the FastPrep-24 Instrument (MP Biomedicals) at 6.0 ms^−1^ for 40 s, according to manufacturer’s instructions. Purified DNA was resuspended in sterile deionized DNAse/pyrogen-free water, analyzed by spectrophotometry (NanoDrop 2000C; Labtech, Heathfield, East Sussex), and frozen (−20 °C) until analysis.

### 4.6. Intracaecal Microbiota Sequencing

Barcoded primers Bact-515F (5′-GTGCCAGCMGCNGCGC-3′) and Bact-1061R (5′-CRRCACGAGCTGACGAC-3′) described by Klindworth et al. (2013) [46] were used for the initial amplification of the V4-V6 region of the 16S rRNA gene as previously described [3]. PCR reactions contained 2.5 U of *Taq* DNA Polymerase (Invitrogen, Cergy Pontoise, France), 5 µL of 5X buffer, 75 nmol MgCl_2_, 1 µL of 10 mM dNTPs, 1 µL of each primer (50 µM) and 50 ng of DNA. Three PCR reactions were run for each sample as follows: 95 °C for 5 min, followed by 40 cycles at 95 °C for 45 s, 60 °C for 45 s, 72 °C for 45 s and a final extension at 72 °C for 5 min. PCR reactions from the same sample were pooled, purified using the QIAquick PCR purification kit (Qiagen, Courtaboeuf, France) and quantified using a Qubit 2.0 Fluorometer (Life Technologies, Carlsbad, CA, USA) using the dsDNA HS Assay Kit (Life Technologies). To ensure equal representation of each sample in the sequencing run, each barcoded sample was standardized by calculating equimolar amounts (100 ng/sample) using the SequalPrep Normalization Plate Kit (Invitrogen) prior to pooling. Pooled samples of the 16S rRNA gene multiplexed amplicons were sequenced on a Roche 454 Genome Sequencer FLX Titanium instrument using the GS FLX Titanium XLR70 sequencing reagents and protocols (Beckman Coulter Genomics, Danvers, MA, USA).

### 4.7. Amplicon Sequencing Data Analysis

Analysis of amplicon sequencing data was carried out using the MEGAN pipeline [47]. After demultiplexing, combined raw sequencing data plus metadata were filtered to exclude low-quality reads. Next, data were denoised and clustered using the MIRA 4 software (http://mira-assembler.sourceforge.net). Sequences with ≥98% similarity were binned and assigned to the same OTU to approximate species-level phylotypes. Representative sequences of each OTU, derived from clusters or singletons, were assigned at different taxonomic level by using the Ribosomal Database Project II Classifier [48]. To avoid a potential bias linked to variation of sequence coverage between samples, the data were normalized to 100,000 sequences per samples. Rarefaction curves were constructed to evaluate sequencing depth. Relative abundances of each OTU were compared according to the different experimental conditions. Bacterial richness and diversity across samples were estimated by calculating the following indexes as previously described [3]: Shannon index, Evenness index, OTU’s number, Simpson’s index of diversity, and Simpson’s reciprocal index. PCA was conducted to appreciate overall distance between microbial communities, using relative abundance and taxa-to-taxa distance estimates. Obtained 16S rRNA gene sequences have been deposited into NCBI’s Sequence Read Archive database (https://www.ncbi.nlm.nih.gov/sra) under accession number SRP153311.

### 4.8. Intracaecal Bacterial Load Quantification

The amount of total bacteria was assessed by amplifying 0.5 ng of DNA extracted from each fecal sample with pan-bacterial primers targeting the 16S rRNA gene as previously described [3]. Briefly, PCR assays were performed using the MESA FAST qPCR MasterMix for SYBRAssay as recommended by the manufacturer (Eurogentec, Seraing, Belgium). DNA extracted from the *Barnesiella intestinihominis* DSM 21032^T^ strain using the QIAamp DNA Mini Kit (Qiagen) was used to establish the standard curves. All assays were performed in triplicate. The following thermocycling conditions were applied with the MyiQ™2 real-time PCR system (Bio-Rad Laboratories, Hercules, CA, USA): initial denaturation at 95 °C for 5 min followed by 40 cycles of 95 °C for 15 s and 60 °C for 1 min. Melting curves were obtained immediately after the amplification under the following conditions: 70 cycles of 10 s with an increment of 0.5 °C/cycle starting at 60 °C.

### 4.9. Statistical Analysis

Comparison of body weights, corticosterone concentrations, normalized thymus weights, bacterial loads quantified by qPCR, relative abundances, and phylogenetic diversity indexes were performed using the Mann–Whitney U test with a significance level α of 0.05. *p*-values comprised between 0.05 and 0.10 indicate trend. The *p*-values were adjusted for multiple hypotheses testing using the False Discovery Rate method [49] for all the results within each taxonomy level. The PERMANOVA analysis (99 permutations) was conducted on dissimilarity indices produced by the Bray–Curtis method [50]. The β-diversity PCA was produced using Marti Anderson’s procedure for the analysis of multivariate homogeneity of group dispersions [51]. All the analysis were performed using R version 3.5.0 (https://www.R-project.org/).

## Figures and Tables

**Table 1:** Comparison of socio-environmental stressors encountered during space missions with those delivered using the CUMS model, and limitations of this model (adapted from [26]).

Socio-Environmental Stressors Applied in the CUMS Model (Details in Figure 1a)	Socio-Environmental Stressors Encountered during Spaceflights	Limitations of CUMS Model
Mice confined in a small cage during 1 or 2 h.	Confinement throughout the mission.	Mice confined during short periods (ethical point of view) /astronauts confined for several months in the ISS.
Isolation of mice (sociable animals) during the whole CUMS procedure.	Isolation from friends and family.	
Reversed light/dark cycle during week-end.	Disrupted circadian rhythm.	Astronauts observe 16 sunrises and sunsets during a 24 h period.
Pair housing during 2 h.	Crew tension and other interpersonal issues.	Pair housing is of a limited duration.
Cage tilt for 1, 2 or 15 h.	Perturbation of spatial references.	Cage tilt is of a limited duration.
Period with difficult access to food, without a reduction in the daily food ration.	Lower dietary intake despite enough available food, changes in eating habits and rituals.	
Period in a soiled cage (mice do not like wet litter).	Uncomfortable living conditions.	

## Supplementary Materials

Supplementary materials can be found at https://www.mdpi.com/1422-0067/21/21/7863/s1. Figure S1. Rarefaction curves of bacterial 16S rRNA gene sequences obtained from (A) control and (B) CUMS mice. These curves were used to evaluate if further sequencing would likely detect additional taxa. Datasets S1: Statistical analysis of intracaecal microbiomes comparing relative abundance using the nonparametric Mann-Whitney U test at the phylum level (Dataset S1_a), at the class level (Dataset S1_b), at the order level (Dataset S1_c), at the family level (Dataset S1_d), at the genus level genera (Dataset S1_e) and at the species level (Dataset S1_f).

## Abbreviations

CMS	chronic mild stress
CUMS	chronic unpredictable mild stress
GM	gut microbiota
HPA	hypothalamic-pituitary-adrenal
IBD	intestinal bowel disease
LDA	linear discriminant analysis
OTUs	operational taxonomic units
PCA	principal component analysis
TCR	T cell receptor
